# Supplementary material for: Seasonal activities of the phyllosphere microbiome of perennial crops
Source: Nat Commun. 2023 Feb 23;14:1039. doi: 10.1038/s41467-023-36515-y (PMC9950430; doi:10.1038/s41467-023-36515-y)
Supplement: Supplementary file 1 — Supplementary Information File [file 41467_2023_36515_MOESM1_ESM.pdf]

Supplementary Information for:

A Howe, N Stopnisek, SK Dooley, F Yang, KL Grady, and A Shade. 2023. Seasonal activities of the phyllosphere microbiome of perennial crops. Nature Communications.

Figure S1.

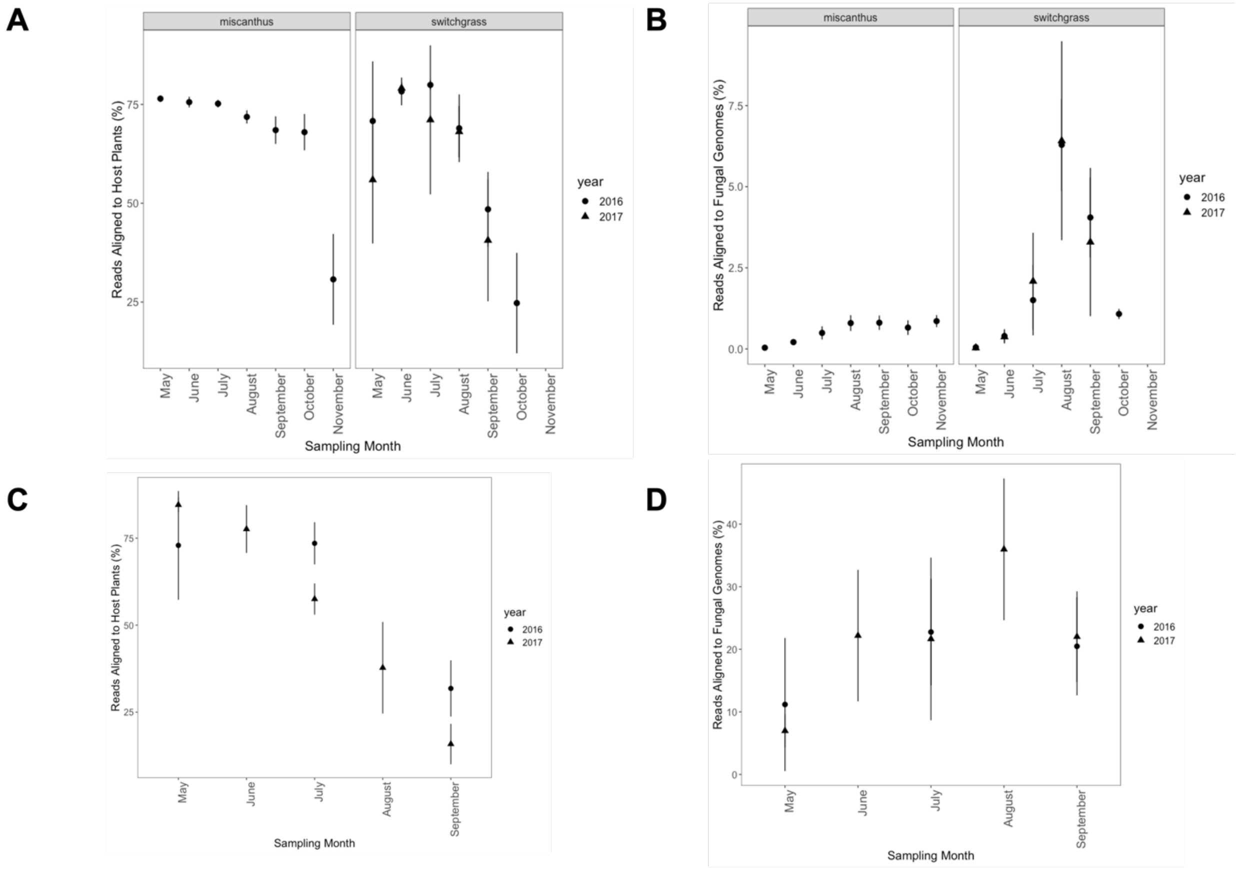

Figure S1. Contaminating plant or fungal sequences. Percent of sequencing in metagenomes originating from miscanthus and switchgrass phyllosphere samples associated with miscanthus and switchgrass host genomes (A) and prevalent fungal genomes (B, Table S1). Percent of sequencing in metatranscriptomes originating from switchgrass phyllosphere samples and associated with switchgrass host genomes (C) and fungal genomes (D, Table S1). Sample sizes are provided in Table 1 and included 64 and 56 metagenomes for switchgrass in 2016 and 2017, respectively, and 72 metagenomes from miscanthus in 2016. Circles are for 2016 and triangles are for 2017. Data are presented as mean values +/- standard deviation. Source data are provided as a Source Data file.

Figure S2

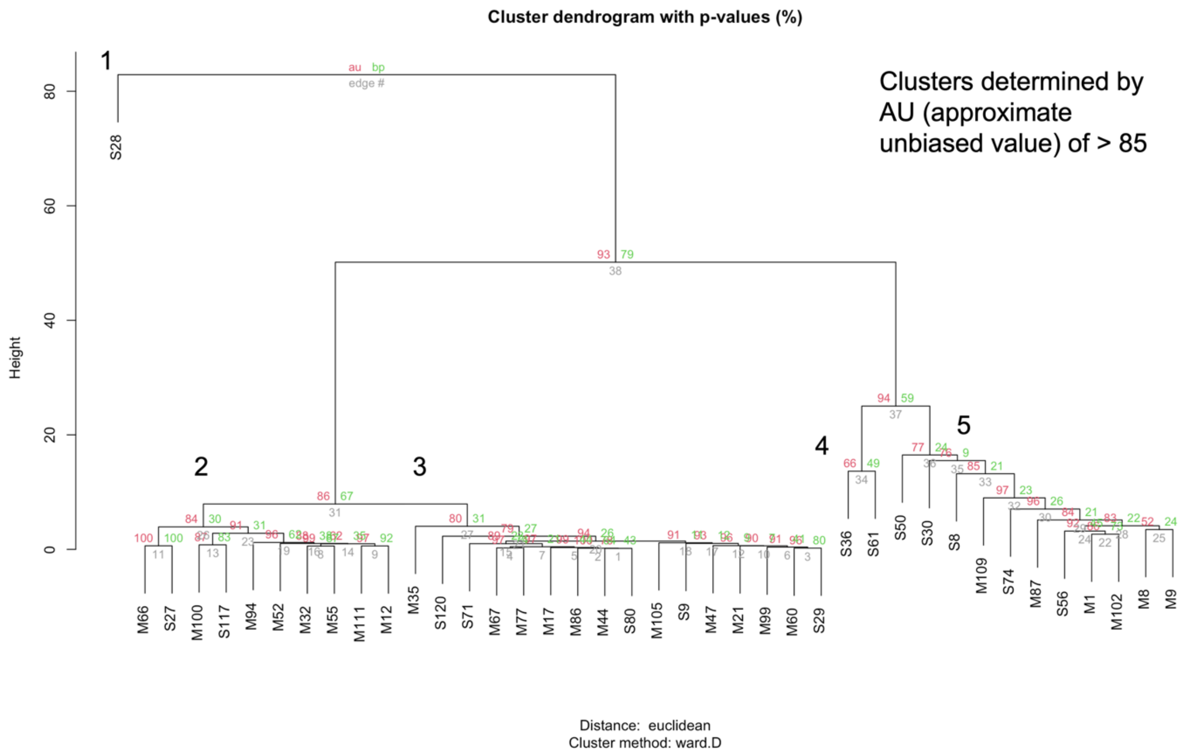

Figure S2. Hierarchical clustering (Ward's method) identified five clusters of MAGS to identify MAG populations with coherent seasonal activity dynamics. Clustering was based on metatranscriptome diversity and abundance. Source data are provided as a Source Data file.

Figure S3

A. 2016 Switchgrass Transcripts

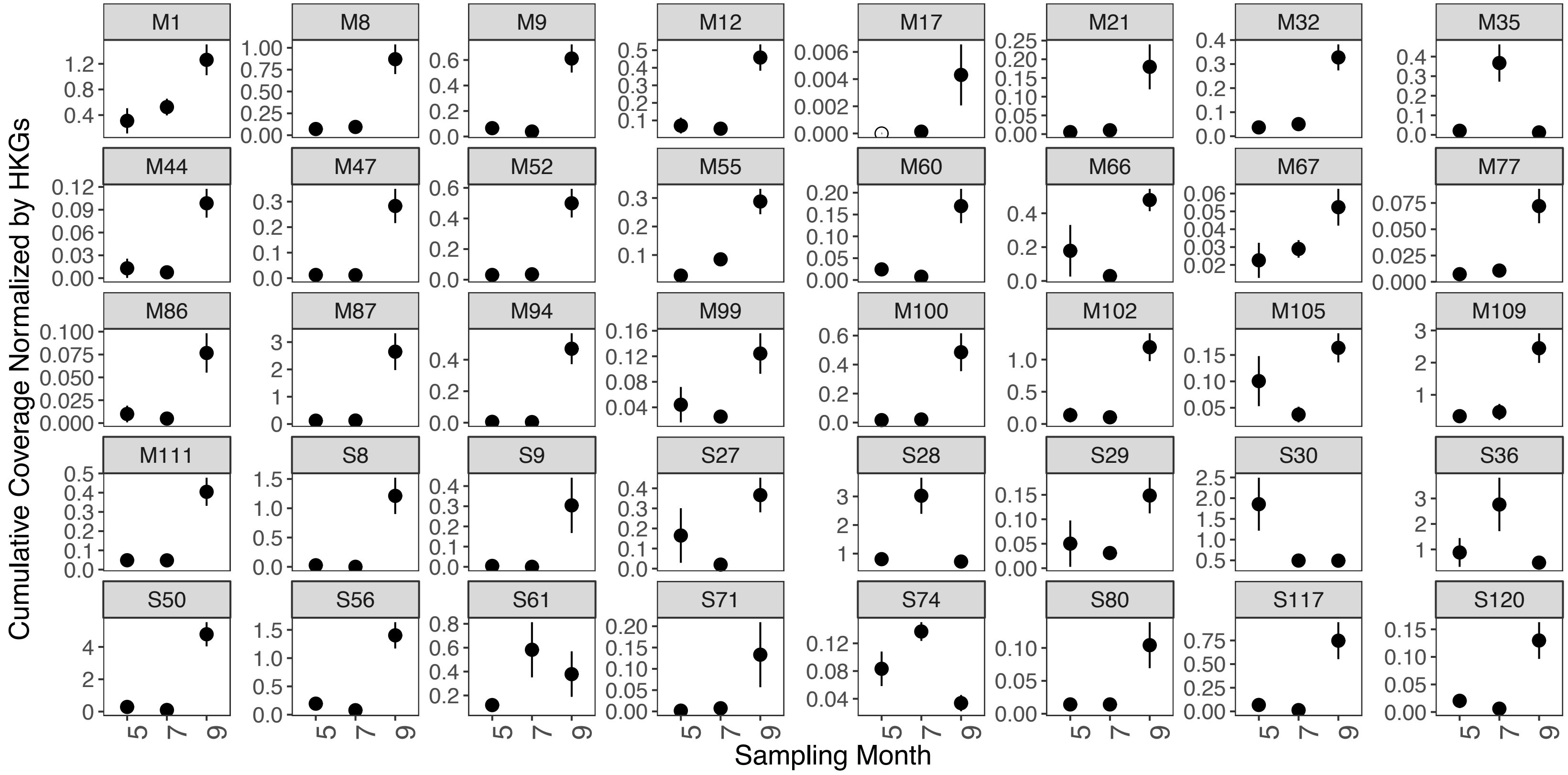

B. 2017 Switchgrass Transcripts

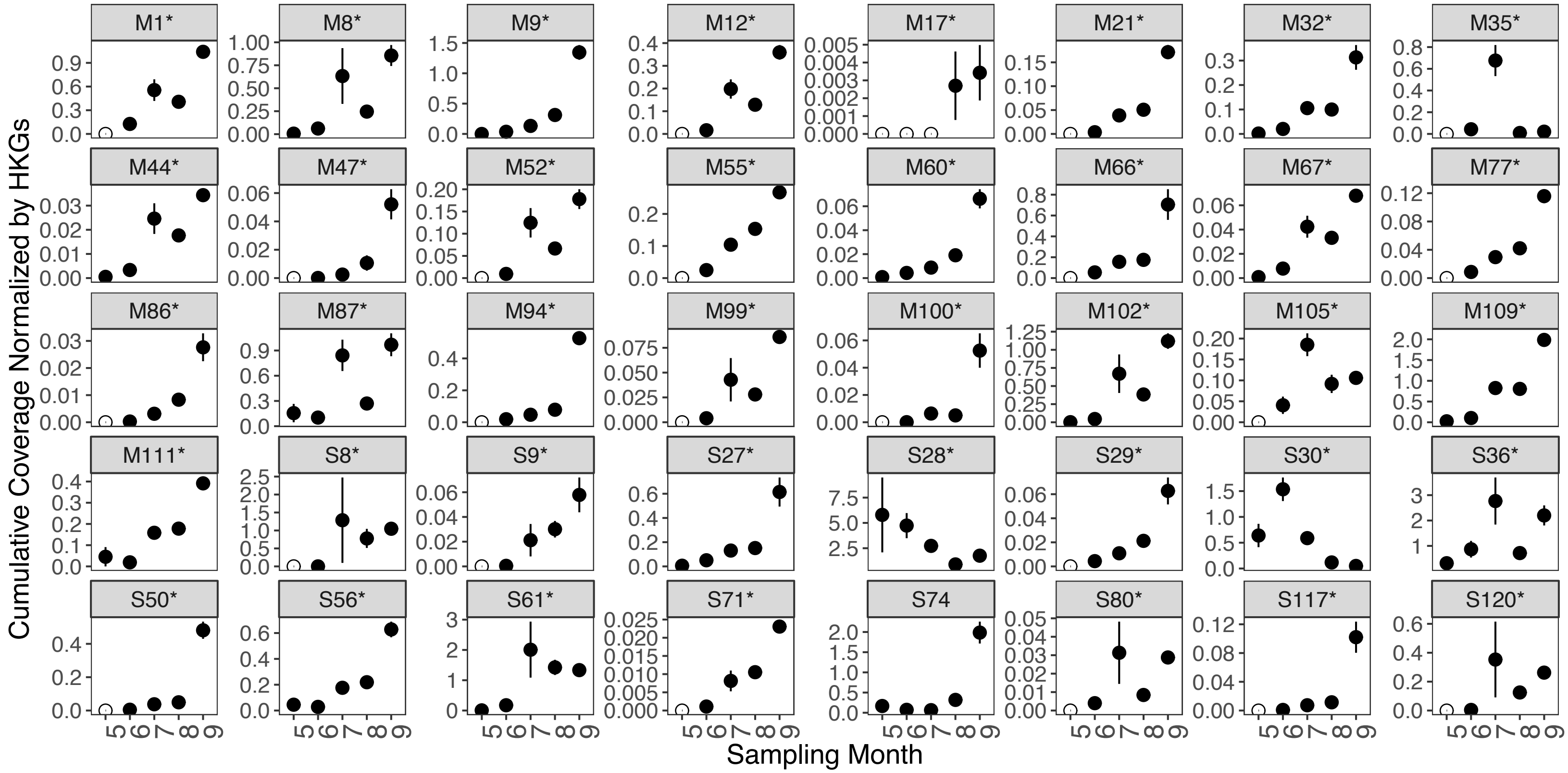

Figure S3. Mean switchgrass leaf transcript abundance by month for each MAG for (A) 2016 and (B) 2017. Note that y-axes vary for each MAG. Filled circles indicate that identification of transcripts and empty circles indicate no detection. Significant statistical differences (two-sided Kruskal-Wallis based on chi-squared distributions, $p < 0.05$) between early (May-June) and late months (July – September) are indicated with asterisks for 2017 transcripts. 2016 transcripts could not be robustly statistically evaluated due to their more limited time series. Sample sizes are provided in Table 1 and included 22 and 56 metatranscriptomes for switchgrass in 2016 and 2017, respectively. Data are presented as mean values +/- standard error of the mean. Source data are provided as a Source Data file.

Exact p-values are as follows: M1 chi-squared = 30.021267, p = 0.00000004273339; M100 chi-squared = 32.377231, p = 0.00000001269654; M102 chi-squared = 36.99858, p = 0.000000001182153; M105 chi-squared = 28.78018, p = 0.00000008107659; M109 chi-squared = 40.116263, p = 0.0000000002392886; M111 chi-squared = 33.038472, p = 0.000000009035318; M12 chi-squared = 39.554385, p = 0.0000000003190492; M17 chi-squared = 9.925781, p = 0.001629797; M21 chi-squared = 38.81277, p =

0.0000000004664662; M32 chi-squared = 36.374657, p = 0.000000001628065; M35 chi-squared = 11.113822, p = 0.0008568675; M44 chi-squared = 35.938939, p = 0.000000002035989; M47 chi-squared = 30.241419, p = 0.00000003814753; M52 chi-squared = 38.826207, p = 0.0000000004632662; M55 chi-squared = 39.974057, p = 0.0000000002573583;M60 chi-squared = 28.871102, p = 0.0000000773585; M66 chi-squared = 29.318955, p = 0.00000006139222; M67 chi-squared = 36.880497, p = 0.00000000125596; M77 chi-squared = 33.650889, p = 0.000000006594473; M8 chi-squared = 33.676723, p = 0.000000006507472; M86 chi-squared = 36.281932, p = 0.000000001707395; M87 chi-squared = 26.005275, p = 0.0000003404858; M9 chi-squared = 36.424744, p = 0.000000001586759; M94 chi-squared = 31.176728, p = 0.00000002355734; M99 chi-squared = 33.354884, p = 0.000000007678475; S117 chi-squared = 38.418137, p = 0.0000000005709925; S120 chi-squared = 35.618369, p = 0.000000002400136; S27 chi-squared = 27.120626, p = 0.0000001911476; S28 chi-squared = 11.859649, p = 0.0005736335; S29 chi-squared = 31.363694, p = 0.00000002139443; S30 chi-squared = 24.671053, p = 0.0000006799787; S36 chi-squared = 13.147204, p = 0.0002879486; S50 chi-squared = 38.466743, p = 0.0000000005569472; S56 chi-squared = 36.194287, p = 0.000000001785934; S61 chi-squared = 36.269636, p = 0.000000001718201; S71 chi-squared = 36.497879, p = 0.000000001528325; *S74 chi-squared = 3.022307, p = 0.08212655 not significant*; S8 chi-squared = 41.405128, p = 0.0000000001237324; S80 chi-squared = 26.576977, p = 0.0000002532437; S9 chi-squared = 30.125029, p = 0.00000004050695

Figure S4

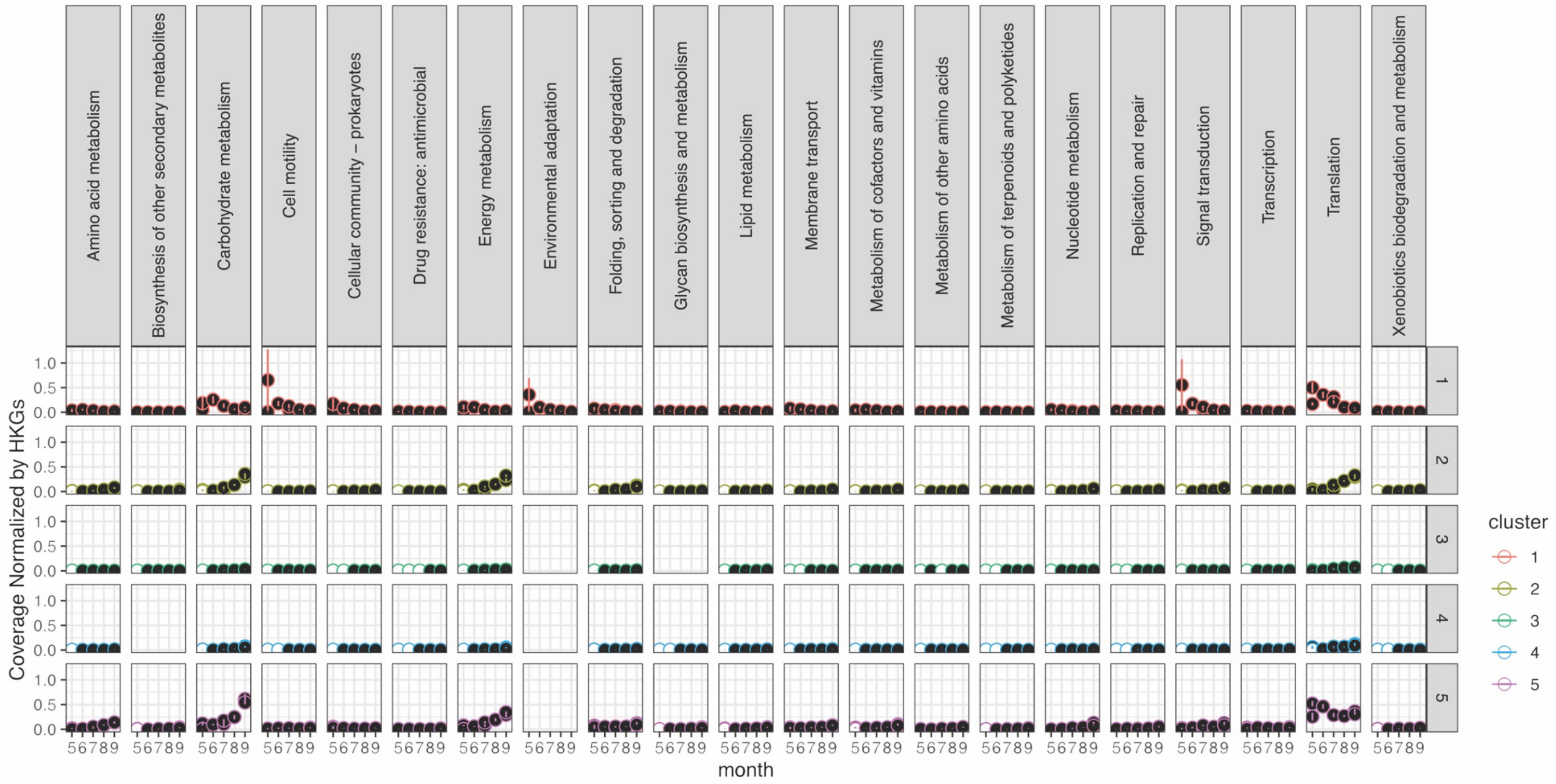

Figure S4. Mean switchgrass leaf transcript dynamics by month and year for each MAG cluster. Filled circles indicate that identification of transcripts and empty circles indicate no detection. Data are presented as mean values +/- standard error of the mean. Source data are provided as a Source Data file.

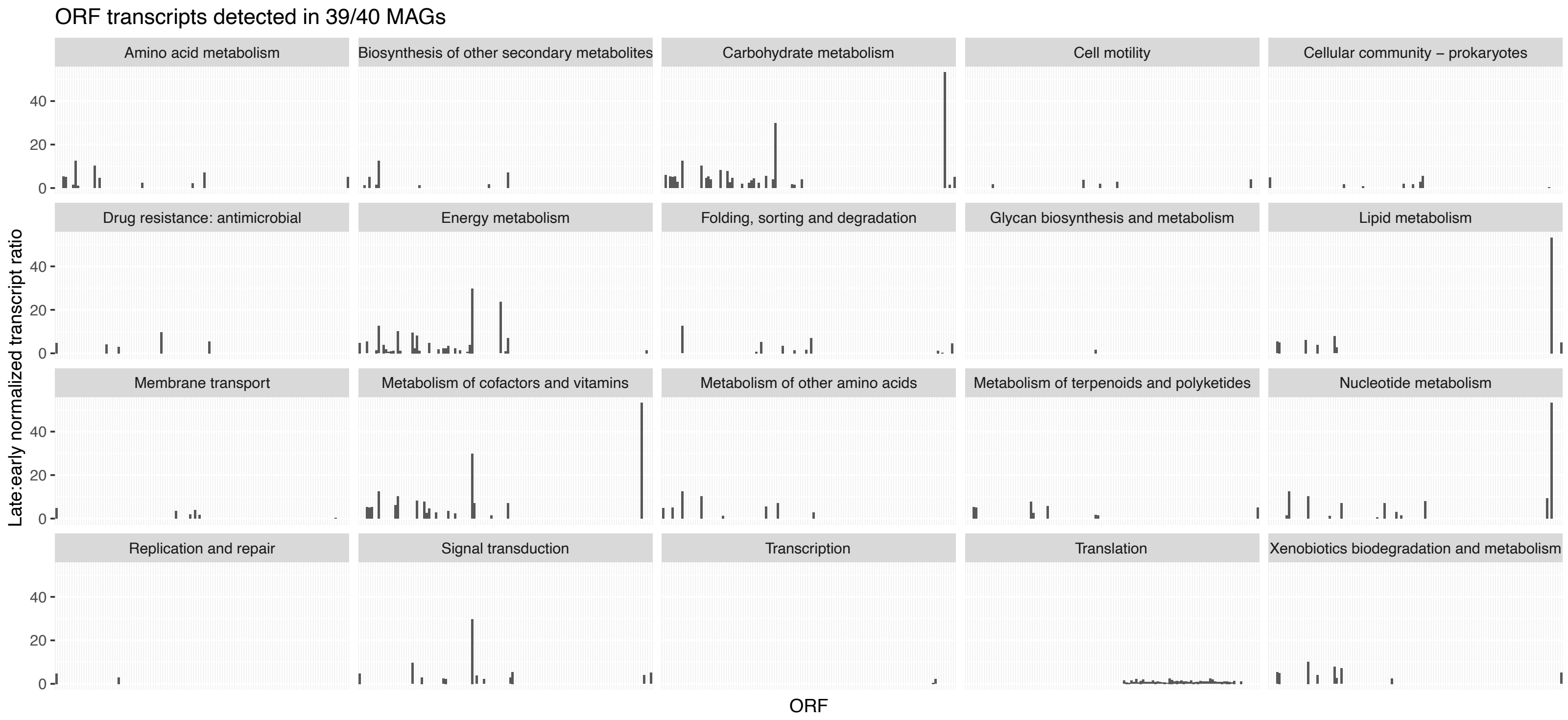

Figure S5. Summary of switchgrass transcript seasonality of phyllosphere open reading frames (ORFs) that could be annotated as KEGG functional roles and were consistently detected among focal MAGs (at least 39/40 detections). Ratios are late-to-early normalized transcript abundances on MAGs. Source data are provided as a Source Data file.

Figure S6

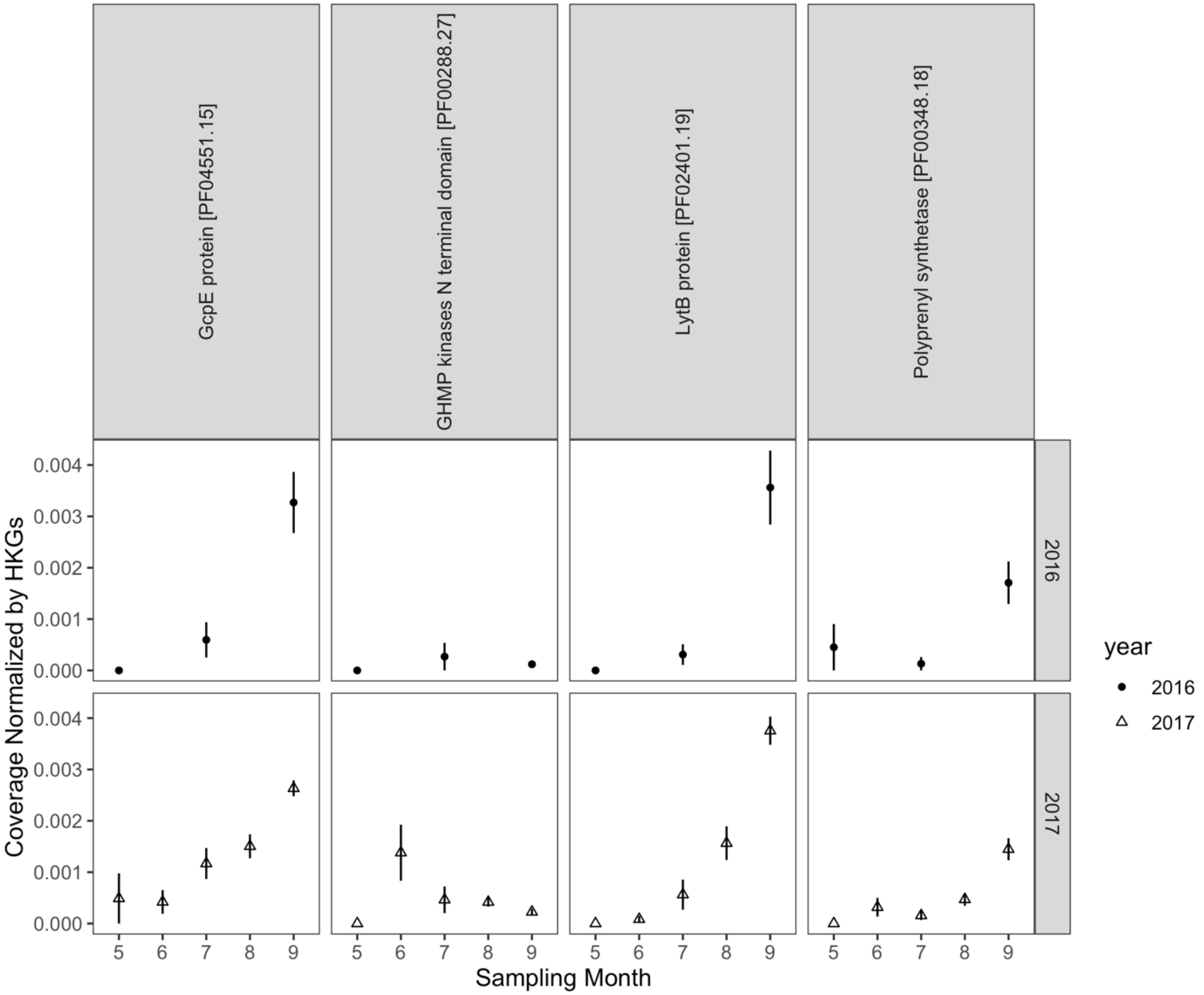

Figure S6. 2016 (circle) and 2017 (triangle) switchgrass leaf transcript dynamics of KEGG metabolism classifications associated with terpenoid backbone biosynthesis. Sample sizes are provided in Table 1. Data are presented as mean values +/- standard error of the mean. Source data are provided as a Source Data file.

Table S1.
Fungal genomes that were used for filtering metagenome reads to remove eukaryotic contamination. Genomes were selected to use for filtering based on the taxonomic identities of prevalent fungal taxa detected in our previous ITS2 amplicon survey that was conducted at the same location and from the same samples (Bowsher *et al.*, 2020).

**Fungal Genome**	**JGI Project ID (*) or GenBank accession (**)**	**Reference**
Trichoderma harzianum	403727*	(Druzhinina et al., 2018)
Pleomassaria siparia	1011309*	(Haridas et al., 2020)
Aureobasidium pullulans	403628*	(Gostinčar et al., 2014)
Alternaria alternata	1103683*	NA
Didymella zeae-maydis	https://genome.jgi.doe.gov/portal/pages/dynamicOrganismDownload.jsf?organism=Didma1	NA
Sporobolomyces roseus	16892*	NA
Puccinia novopanici	GCA_004348175.1**	(Gill et al., 2019)

Table S2. Two-sided linear regression analysis without adjustments to evaluate the relationship of cumulative transcript abundances in KEGG metabolic classes and sampling month. Bold indicates p-values < 0.05.

	Slope	R^2	p-value
**Amino acid metabolism**	**0.048**	**0.51**	**2.4E-13**
**Biosynthesis of other secondary metabolites**	**0.012**	**0.48**	**2.7E-12**
**Carbohydrate metabolism**	**0.183**	**0.43**	**1.2E-10**
Cell motility	-0.075	0.03	1.0E-01
**Cellular community - prokaryotes**	**-0.021**	**0.07**	**1.9E-02**
Drug resistance: antimicrobial	0.002	0.03	1.4E-01
**Energy metabolism**	**0.133**	**0.55**	**1.2E-14**
Environmental adaptation	-0.047	0.04	6.5E-02
**Folding, sorting and degradation**	**0.030**	**0.26**	**2.4E-06**
**Glycan biosynthesis and metabolism**	**0.006**	**0.18**	**9.7E-05**
**Lipid metabolism**	**0.014**	**0.37**	**3.5E-09**
**Membrane transport**	**0.013**	**0.07**	**1.6E-02**
**Metabolism of cofactors and vitamins**	**0.020**	**0.20**	**3.7E-05**
**Metabolism of other amino acids**	**0.015**	**0.50**	**9.2E-13**
**Metabolism of terpenoids and polyketides**	**0.007**	**0.49**	**1.8E-12**
**Nucleotide metabolism**	**0.031**	**0.40**	**6.1E-10**
**Replication and repair**	**0.014**	**0.27**	**1.5E-06**
Signal transduction	-0.036	0.01	3.5E-01
**Transcription**	**0.004**	**0.09**	**8.2E-03**
Translation	0.004	0.00	7.6E-01
**Xenobiotics biodegradation and metabolism**	**0.012**	**0.47**	**8.5E-12**

Table S3.
Summary of the nine genes involved in the *Bacillus subtilus* isoprene biosynthesis pathway that were detected among focal MAG contigs. Except for *ypgA*, these genes were directly linked to isoprene accumulation (Julsing *et al.*, 2007).

Gene	*dxr*	*dxs*	*gcpE*	*idi*	*lytB*	*yacM*	*ychB*	*ygbB*	*yqiD*
NCBI Acc. No.	NP_389537.2	NP_390307.1	NP_390386.1	BAB32625.1	NP_390395.2	NP_387971.1	NP_387927.1	NP_387972.1	NP_390308.2
M94	+	+	+	+	+		+	+	+
S30	+	+	+	+	+		+	+	+
M105	+	+	+		+		+	+	+
M12	+	+	+	+	+			+	+
M32	+	+	+		+		+	+	+
M52	+	+	+	+	+			+	+
M67	+	+	+		+	+	+		+
M77	+	+	+		+		+	+	+
S117		+	+	+	+		+	+	+
S28	+	+	+		+		+	+	+
S56	+	+	+	+			+	+	+
M100	+	+	+		+	+			+
M109	+	+	+		+			+	+
M1	+	+			+		+	+	+
M21	+	+	+		+		+		+
M44	+	+		+	+		+		+
M47		+	+		+	+		+	+
M60		+	+		+		+	+	+
M66	+	+	+				+	+	+
M86	+	+	+		+		+		+
M8		+	+		+		+	+	+
M99	+	+			+	+		+	+
M9	+	+	+		+			+	+
S27	+	+	+		+			+	+
S61	+	+	+		+			+	+
S71	+	+			+		+	+	+
S74	+	+	+		+	+			+
M102	+	+	+					+	+
M55	+				+		+	+	+
S80		+	+		+	+			+
M111			+	+	+				+
M87	+	+						+	+
S120		+	+		+		+		
S29		+					+	+	+
S50	+	+	+				+		
S36		+	+		+				
M17		+							+
M35		+							+
S8		+							+
S9		+							+
