# Supplementary material for: Seasonal activities of the phyllosphere microbiome of perennial crops
Source: Nat Commun. 2023 Feb 23;14:1039. doi: 10.1038/s41467-023-36515-y (PMC9950430; doi:10.1038/s41467-023-36515-y)
Supplement: Supplementary file 3 — Description of Additional Supplementary Files [file 41467_2023_36515_MOESM3_ESM.pdf]

Description of Additional Supplementary Files:

Supplementary Data 1. Metadata for metagenome samples (switchgrass and miscanthus) according to MIMs standards. Includes environmental contextual data as well as metadata about sequencing protocols, returned reads, and bioproject identifiers.

Supplementary Data 2. Metadata for metatranscriptome samples (switchgrass) according to MIMs standards. Includes environmental contextual data as well as metadata about sequencing protocols, returned reads, and bioproject identifiers.

Supplementary Data 3. Sheet 1: Metadata for selected focal 40 metagenome-assembled genomes (>50% complete, <10% contamination) according to MiMAG standards. Sheet 2: Other MAGs (non-focal) that were assembled from switchgrass or miscanthus metagenomes. Occupancy is the proportion of the 192 metagenomes in which a MAG was detected, and abundance is the average normalized by housekeeping gene (HKG) MAG coverage across the 192 metagenomes.

Supplementary Data 4. Abundances of MAG contigs in metagenomes based on median base pair of read recruitment divided by the average median base pair coverage of housekeeping genes.

Supplementary Data 5. Abundances of MAG open reading frames (ORFs) in metatranscriptomes based on the median basepair coverage of all reads mapped to ORFs and divided by the median basepair coverage of housekeeping genes.

Supplementary Data 6. Sheet 1: Summary of the metagenome reads that mapped to the focal MAGs. Total QC reads are the number of reads after trimming for quality scores, adapters, and removal of host- and fungal-associated sequences. Sheet 2: Summary of metatranscriptome reads that mapped to the focal MAGs. Total QC reads are the number of reads after trimming for quality scores, adapters, and removal of host and fungal-associated sequences.

Supplementary Data 7. Publicly available soil metagenomes from corn, switchgrass, and miscanthus cropping systems in Michigan, Iowa, and Wisconsin obtained via the Integrated Microbial Genomes (IMG) database. Additionally, see Figure 8, which estimates the abundances of MAGs in these metagenomes.
