# Supplementary material for: Seasonal activities of the phyllosphere microbiome of perennial crops
Source: Nat Commun. 2023 Feb 23;14:1039. doi: 10.1038/s41467-023-36515-y (PMC9950430; doi:10.1038/s41467-023-36515-y)
Supplement: Supplementary file 11 — Reporting Summary [file 41467_2023_36515_MOESM11_ESM.pdf]

nature portfolio

Corresponding author(s): Shade, Ashley

Last updated by author(s): Jan 23, 2023

Reporting Summary

Nature Portfolio wishes to improve the reproducibility of the work that we publish. This form provides structure for consistency and transparency in reporting. For further information on Nature Portfolio policies, see our Editorial Policies and the Editorial Policy Checklist.

Statistics

For all statistical analyses, confirm that the following items are present in the figure legend, table legend, main text, or Methods section.

n/a	Confirmed	
☐	☒	The exact sample size (*n*) for each experimental group/condition, given as a discrete number and unit of measurement
☐	☒	A statement on whether measurements were taken from distinct samples or whether the same sample was measured repeatedly
☐	☒	The statistical test(s) used AND whether they are one- or two-sided<br>*Only common tests should be described solely by name; describe more complex techniques in the Methods section.*
☒	☐	A description of all covariates tested
☒	☐	A description of any assumptions or corrections, such as tests of normality and adjustment for multiple comparisons
☐	☒	A full description of the statistical parameters including central tendency (e.g. means) or other basic estimates (e.g. regression coefficient) AND variation (e.g. standard deviation) or associated estimates of uncertainty (e.g. confidence intervals)
☐	☒	For null hypothesis testing, the test statistic (e.g. *F*, *t*, *r*) with confidence intervals, effect sizes, degrees of freedom and *P* value noted<br>*Give P values as exact values whenever suitable.*
☒	☐	For Bayesian analysis, information on the choice of priors and Markov chain Monte Carlo settings
☒	☐	For hierarchical and complex designs, identification of the appropriate level for tests and full reporting of outcomes
☒	☐	Estimates of effect sizes (e.g. Cohen's *d*, Pearson's *r*), indicating how they were calculated

Our web collection on statistics for biologists contains articles on many of the points above.

Software and code

Policy information about availability of computer code

Data collection	Illumina HiSeq 2500, Illumina NovaSeq 6000
Data analysis	Trimmomatic (v0.39), bowtie2 (v 2.4.1),samtools (v 1.13), and bedtools (v2.30.0), MEGAHIT (v 1.2.9), Metabat (v2.2.15), CheckM (v.1.13), dRep (v3.2.0), Prodigal (v2.6.3), DRAM tool (v1.1.1), [UniRef90, MEROPS, PFAM, dbCAN-HMMdb (v8), compiled 12 Feb 2021], KEGG release 01 January 2018, GTDB-tk (v1.4.0), BLAST (2.10.1), R environment for statistical computing releases from 2021-2022 (pvclust version 2.20), antiSMASH (v6.0), Big-SCAPE (v1.1.0) , gapseq with MetaCyc (2021)<br><br>GitHub code is available: https://github.com/ShadeLab/PAPER_Howe_2021_switchgrass_MetaT; https://zenodo.org/record/10040

For manuscripts utilizing custom algorithms or software that are central to the research but not yet described in published literature, software must be made available to editors and reviewers. We strongly encourage code deposition in a community repository (e.g. GitHub). See the Nature Portfolio guidelines for submitting code & software for further information.

Data

Policy information about availability of data

All manuscripts must include a data availability statement. This statement should provide the following information, where applicable:

- Accession codes, unique identifiers, or web links for publicly available datasets
- A description of any restrictions on data availability
- For clinical datasets or third party data, please ensure that the statement adheres to our policy

Data Availability
The raw and processed metagenome and metatranscriptome data generated in this study have been deposited in the Joint Genome Institute Genome Portal database under proposal ID 503249 [https://genome.jgi.doe.gov/portal/Seadynanfunction/Seadynanfunction.info.html]. The MAGs data generated in this study have been depositied in NCBI under bioproject PRJNA800073 [https://www.ncbi.nlm.nih.gov/bioproject/PRJNA800073]. The metadata, including metadata standards for metagenomes, metatranscriptomes, and metagenome-assembled genomes, for this study are provided in the Supplementary Data 1-8. Source data are provided with this paper.

Code Availability
Annotated code and links to data and metadata are available on GitHub (https://github.com/ShadeLab/PAPER_Howe_2021_switchgrass_MetaT) and https://zenodo.org/record/10040 .

Human research participants

Policy information about studies involving human research participants and Sex and Gender in Research.

Reporting on sex and gender	NA
Population characteristics	NA
Recruitment	NA
Ethics oversight	NA

Note that full information on the approval of the study protocol must also be provided in the manuscript.

Field-specific reporting

Please select the one below that is the best fit for your research. If you are not sure, read the appropriate sections before making your selection.

☐ Life sciences ☐ Behavioural & social sciences ☒ Ecological, evolutionary & environmental sciences

For a reference copy of the document with all sections, see nature.com/documents/nr-reporting-summary-flat.pdf

Ecological, evolutionary & environmental sciences study design

All studies must disclose on these points even when the disclosure is negative.

Study description

Site description and sampling scheme
Switchgrass and miscanthus leaves and corresponding contextual data were collected at the Great Lakes Bioenergy Research Center (GLBRC) located at the Kellogg Biological Station (KBS) in Hickory Corners, MI, USA (42o23'41.6" N, 85o22'23.1" W) (Figure 1). We sampled switchgrass (Panicum virgatum L. cultivar Cave-in-rock) and miscanthus (Miscanthus x giganteus) from the Biofuel Cropping System Experiment (BCSE) sites, plot replicates 1-4, as previously described (Grady et al., 2019). This included collecting leaves from switchgrass and miscanthus at eight and nine time points, respectively, in 2016 and switchgrass at seven time points in the 2017 season (Table 1, Figure 1, Dataset 1, Dataset 2). We collected leaves for RNA isolation at three phenology-informed switchgrass time points in 2016 (emergence, peak growth, and senescence) according to GLBRC standard phenology methods https://data.sustainability.glbrc.org/protocols/165) to assess the potential for sufficient mass and quality RNA extraction from the switchgrass leaf surface, and then expanded to include leaves from all switchgrass sampling time points in 2017. Leaves for RNA isolation were flash-frozen in liquid nitrogen immediately and stored at -80oC until processing. See Figure 1 for temporal-spatial study design and Table 1 for returned reads per sample.

This was an observational study. There were no active experimental treatments or interactions. The field design as established by the long term ecological research site was a randomized block. There were 4 replicate blocks per crop observed over time.

Table 1. Summary of RNA and DNA samples that returned reads and passed Illumina standard quality control at the Joint Genome Institute. The total collected samples submitted for sequencing is provided first, and the number of quality sequencing datasets returned for analysis is given in parentheses.

	Metagenome 2016 /Metatranscriptome2016 /Metagenome 2017 /Metatranscriptome 2017<br><br>Switchgrass: 8 time points (64/64 successful)/ 3 time points (22/24 successful) /7 time points (56/56 successful) /7 time points (56/56 successful)<br>Miscanthus: 9 time points (72/72 successful) /Not assessed /Not assessed /Not assessed
Research sample	Four each of miscanthus and switchgrass plots in the KBS LTER BCSE, sampled over time. Two seasons for switchgrassa nd one for miscanthus. Each sample represents a representative microbiome associated to a field of crop of switchgrass or miscanthus grown int he midwestern united states. The data are metagenomes sequenced from nucleic acids extracted/sourced from leaf surfaces of crops grown in the field.
Sampling strategy	We collected leaves for RNA isolation at three phenology-informed switchgrass time points in 2016 (emergence, peak growth, and senescence) according to GLBRC standard phenology methods https://data.sustainability.glbrc.org/protocols/165). Field sample sizes were chosen to capture replicate time series of each crop at key phenological points in a growing season. Field samples were aligned with ongoing LTER work at this site. The sample size was chosen because it captured the dominant known seasonal drivers of microbiome dynamics as informed in our previous study and work in this system (Grady et al. 2019).
Data collection	Leaves and soil were collected and pooled at three flagged sites distributed across each plot, and contextual data was measured at the first flag. Researchers followed a designated walking path to minimize disruption to the ultimate crop yield. A map of the walking path is available with the major LTER protocols: https://lter.kbs.msu.edu/maps/images/glbrc-station-flags.pdf . Field sampling parameters were recorded in a field note book at the time of sampling, and then transferred into digital notes in LabGuru. The PI, post-doctoral associates, graduate students, undergraduate students, and the research technician on the project contributed to data collection and field work and recorded the data. Keara Grady, an author on the paper, was present for field collection throughout the study to ensure consistency and protocol adherence. Every 3 weeks during the 2016 growing season, leaves and soil from switchgrass and miscanthus plots will be collected from the GLBRC Biofuel Cropping System Experiment. This experiment outlines the sample collection strategies that will be used, as well as any sample collection or processing notes<br>http://lter.kbs.msu.edu/research/long-term-experiments/glbrc-intensive-experiment/<br><br>Samples collected are from treatment G5 (switchgrass) reps 1-4 and treatment G6 (miscanthus) reps 1-4. Rep 5 was collected for the first few samples, but has been discontinued due to unexplainable abnormalities in the leaves and plot (per Phil Robertson)<br><br>Contextual data recorded at the time of sample collection by field research assistants (PI, technician, post-docs, graduate students, undergraduate students):<br><br>Soil temperature<br>Air temperature<br>Time of sampling<br>Weather<br>Number of cores taken (not applicable to this study, which is on leaves)<br>Number of leaves<br>Max plant height<br><br>Change gloves and clean corers with ethanol between plots<br>Store samples on ice during transport<br>Switchgrass: Following designated walking path, collect ten leaves per flag, for a total of 30 leaves per plot. Pool in a single bag per block.<br>Miscanthus: Following designated walking path, collect five leaves per flag, for a total of 15 leaves per plot. Pool in a single bag per plot.
Timing and spatial scale	There were four replicate fields for each of two perennial crops. This included collecting leaves from switchgrass and miscanthus at eight and nine time points, respectively, in 2016 and switchgrass at seven time points in the 2017 season (Table 1, Figure 1, Dataset 1, Dataset 2). We collected leaves for RNA isolation at three phenology-informed switchgrass time points in 2016 (emergence, peak growth, and senescence) according to GLBRC standard phenology methods https://data.sustainability.glbrc.org/protocols/165)
Data exclusions	One MAG that had originally met completeness and contamination criteria, M22, had an average of 48 reads map to metatranscriptome and was then removed from further analysis
Reproducibility	This is an observational field study including four replicate plots per crop. All data and code are available https://github.com/ShadeLab/PAPER_Howe_2021_switchgrass_MetaT to reproduce the analysis.
Randomization	At the design of the BCSE (which was a decade before we arrived), a complete randomized block design for the cropping systems' plantings was chosen as appropriate for agricultural field research. Please see the history of the site here: https://lter.kbs.msu.edu/research/long-term-experiments/glbrc-intensive-experiment/
Blinding	Blinding was not used in this study by the researchers, but the sequencing center had no a priori knowledge of the design. There is no active treatment in this study - it is observational.

Did the study involve field work? ☒ Yes ☐ No

Field work, collection and transport

Field conditions	This information is available at the Kellogg Biological Station Long Term Ecological Research Data Catalogue, as well as within the Great Lakes Bioenergy Research Center data catalogue. In addition, the relevant metadata from field conditions are reported in Datasets 1 and 2 as part of the contextual information. The field conditions were previously reported in Grady et al. Nature Communications 2019.
Location	Great Lakes Bioenergy Research Center (GLBRC) located at the Kellogg Biological Station (KBS) in Hickory Corners, MI, USA (42o23'41.6" N, 85o22'23.1" W)
Access & import/export	These are long-term field sites associated with the KBS and the GLBRC (>10 years old) and as investigators associated the Center and researchers at Michigan State University (the institution who "owns" the Kellogg Biological Station), we have field access. No special permits were required. Site access is available to researchers who inquire. https://lter.kbs.msu.edu/who-we-help/prospective-researchers-and-students/
Disturbance	There was no disturbance applied in this study, and understanding disturbance ecology was not a study objective. It was an observational study of typical growing seasons for two crops in the midwest.

Reporting for specific materials, systems and methods

We require information from authors about some types of materials, experimental systems and methods used in many studies. Here, indicate whether each material, system or method listed is relevant to your study. If you are not sure if a list item applies to your research, read the appropriate section before selecting a response.

Materials & experimental systems

n/a	Involved in the study
☒	☐ Antibodies
☒	☐ Eukaryotic cell lines
☒	☐ Palaeontology and archaeology
☒	☐ Animals and other organisms
☒	☐ Clinical data
☒	☐ Dual use research of concern

Methods

n/a	Involved in the study
☒	☐ ChIP-seq
☒	☐ Flow cytometry
☒	☐ MRI-based neuroimaging
